# The Prevalence and Predictors of Hypertension and Albuminuria in People with HIV (PWHIV)—Real-World Greek Data

**DOI:** 10.3390/life15111747

**Published:** 2025-11-13

**Authors:** Vasileios Petrakis, Andreas G. Tsantes, Petros Rafailidis, Konstantia Kantartzi, Maria Panopoulou, Theocharis Konstantinidis, Nikoleta Babaka, Pelagia Kriki, Stylianos Panagoutsos, Dimitrios Papazoglou, Periklis Panagopoulos

**Affiliations:** 1 2nd University Department of Internal Medicine, Department of Infectious Diseases, University General Hospital Alexandroupolis, Democritus University Thrace, 68100 Alexandroupolis, Greece; prafaili@med.duth.gr (P.R.); mpampakanikoleta@gmail.com (N.B.); dpapazog@med.duth.gr (D.P.); ppanago@med.duth.gr (P.P.); 2 Laboratory of Haematology and Blood Bank Unit, “Attiko” Hospital, School of Medicine, National and Kapodistrian University of Athens, 12462 Athens, Greece; 3 Microbiology Department, “Saint Savvas” Oncology Hospital, 11522 Athens, Greece; 4 University Nephrology Department, University General Hospital Alexandroupolis, Democritus University Thrace, 68100 Alexandroupolis, Greece; kkanatart@med.duth.gr (K.K.); pelkriki@gmail.com (P.K.); spanagou@med.duth.gr (S.P.); 5 University Laboratory Department, University General Hospital Alexandroupolis, Democritus University Thrace, 68100 Alexandroupolis, Greece; mpanopou@med.duth.gr (M.P.); tkonsta@med.duth.gr (T.K.)

**Keywords:** HIV, AIDS, hypertension, albuminuria, cardiovascular disease, renal disease

## Abstract

Background: After the implementations of Highly Active Antiretroviral Therapy (HAART) HIV infection became a chronic condition and the clinical focus on non-AIDS-related comorbidities such as hypertension and chronic kidney disease has increased. This study aims to investigate the prevalence and independent predictors of hypertension and albuminuria in a cohort of people with HIV (PWHIV) with high rates of viral suppression. Methods: This is a cross-sectional study of 183 HAART-experienced PWHIV. Hypertension, defined as office systolic blood pressure of ≥140 mmHg or diastolic blood pressure of ≥90 mmHg and albuminuria, was defined as a sex-based albumin–creatinine ratio (ACR) of >355 mg/g for females and >250 mg/g for males. Univariable and multivariable logistic regression was conducted to identify the association of hypertension and albuminuria with demographic, clinical, and HIV-specific factors. Results: The prevalence of hypertension was 43.9% (*n* = 74) and albuminuria was 22.4% (*n* = 41). In the multivariable analysis, factors independently associated with prevalence of HTN were older age, overweight/obesity, and diabetes mellitus. TDF-based ART was explored as a potential factor but did not reach statistical significance (aRR = 1.85, *p* = 0.065). For albuminuria, older age, diabetes mellitus, and duration of HAART (aRR = 1.03 per year) were revealed as independent predictors. Conclusions: The results of this study demonstrate that the development of hypertension is primarily driven by traditional metabolic risk factors. However, the progression to albuminuria appears to be influenced not only by these comorbidities but also by long-term HIV disease and HAART exposure. These findings underline the critical need for the screening and management of hypertension and other comorbidities to mitigate the risk of long-term cardiovascular and renal complications in this aging population of PWHIV.

## 1. Introduction

The global HIV pandemic has been one of the most significant public health challenges of the past century [1]. The advent and remarkable progress of Highly Active Antiretroviral Therapy (HAART) has profoundly altered the natural history of the Human Immunodeficiency Virus (HIV) infection which has been converted from a fatal illness into a manageable, chronic condition [1]. Life expectancy for people with HIV (PWHIV) fully compliant to HAART closely approximates that of the general population [2]. This success has led to a paradigm shift in clinical care with increasing focus on non-AIDS defining conditions and on comorbidities including cardiovascular and renal disease [2]. Among comorbidities, hypertension, albuminuria, and chronic kidney disease represent clinically significant and prevalent conditions, contributing substantially to morbidity in people with HIV (PWHIV) [3]. The improved survival of PWHIV receiving HAART is associated with a heightened risk of developing traditional hypertension risk factors, such as advancing age, lifestyle-related risk factors, and comorbid medical conditions [3,4,5,6]. Hypertension (HTN), a major risk factor for cardiovascular disease, is a growing problem among PWHIV, especially compared with people without HIV. The prevalence of hypertension among PWHIV varies but is documented to be higher in sub-Saharan Africa where the burden of HIV is the highest [4,5]. Concurrently, HIV-associated kidney disease has emerged as a significant cause of morbidity [5]. One of the earliest and most common markers of renal dysfunction is albuminuria, which is an independent predictor not only for renal disease but also for possible cardiovascular events in the future [6]. The co-occurrence of hypertension and albuminuria in PWHIV accelerates the risk of both cardiovascular and kidney disease [6].

The pathophysiological links among HIV infection, HAART, hypertension, and albuminuria are complex and not yet fully understood [7,8,9]. Chronic inflammation, persistent immune activation even in virologically suppressed individuals, metabolic dysregulation and effects of the virus, viral–host interactions, and different treatment options contribute to the increased frequency of comorbidities in PWHIV due to endothelial dysfunction, arterial stiffness, and a pro-coagulant state [7,8,9]. Targeted prevention and management strategies are crucial to be developed in order to reduce the morbidity rates and improve the quality of life highlighting the need of a holistic approach of care beyond viral suppression [10]. Despite the vast body of HIV research, a critical evidence gap exists concerning the precise burden and underlying risk stratification of non-AIDS defining conditions within specific Southern European healthcare settings. A study in Greek population based on cross-sectional comparison of PWHIV (Athens Multicenter AIDS Cohort-Study; AMACS) versus general population controls (National health examination survey; EMENO) showed that the prevalence of hypertension adjusted for age, sex, and country of origin was similar in PWHIV and in the general population (38% vs. 37.6%) [11]. However, after further adjustment for BMI, a known significant predictor of hypertension, the study concluded that PWHIV had higher prevalence of hypertension, compared to the general population [11]. The prevalence of HTN has been found to be increasing throughout the years based on published data in a Greek cohort of PWHIV [12]. Average systolic and diastolic pressure levels were similar during the period 2003–2013, but the proportion of individuals receiving anti-hypertensive treatment increased from 2.2% in 2003 to 3.0% in 2013 (*p* < 0.001) and the proportion of individuals classified as hypertensive increased significantly (*p* < 0.001) from 30.6% in 2003 to 34.4% in 2013 [12]. In a retrospective analysis of the data of 558 newly diagnosed antiretroviral-naïve PWHIV between January 1998 and December 2008 in Northern Greece (Thessaloniki), the prevalence of hypertension was found 33.0% [13].

Despite the vast body of HIV research, a critical evidence gap concerning the precise burden and underlying risk stratification of non-AIDS defining conditions within specific Southern European healthcare settings exists. This study provides new, essential real-world data that close this gap by focusing on a cohort of ART-experienced PWHIV in Northern Greece. The objective of this article is to provide a comprehensive analysis of the prevalence, risk factors, and clinical implications of hypertension and albuminuria in PWHIV monitored in the HIV Unit of University General Hospital of Alexandroupolis. The HIV Unit of Alexandroupolis is located in a rural region with population heterogeneity and a strategic position between West and East, Europe and Asia [14]. Thus, Alexandroupolis is the intermediate station for many refugees or immigrants in order to pass to their final destination, other European countries, and they do not remain in our unit for monitoring [14]. The proportion of PWHIV in our unit diagnosed late is 49.5% and 34% and they developed advanced HIV infection [14]. The majority are males in the age group 31–40 years and men who had sex with men [14]. The notably high percentage of late presenters in our unit demonstrates that late presentation remains a challenge for public health [14]. We aim to explore the demographic, clinical, and laboratory characteristics associated with these conditions and to evaluate the impact of different ART regimens on their development and progression. Further understanding of these critical issues will provide more appropriate knowledge in order to implement effective preventive and therapeutic strategies to enhance the quality of life of people with HIV.

## 2. Materials and Methods

**Figure 1:**
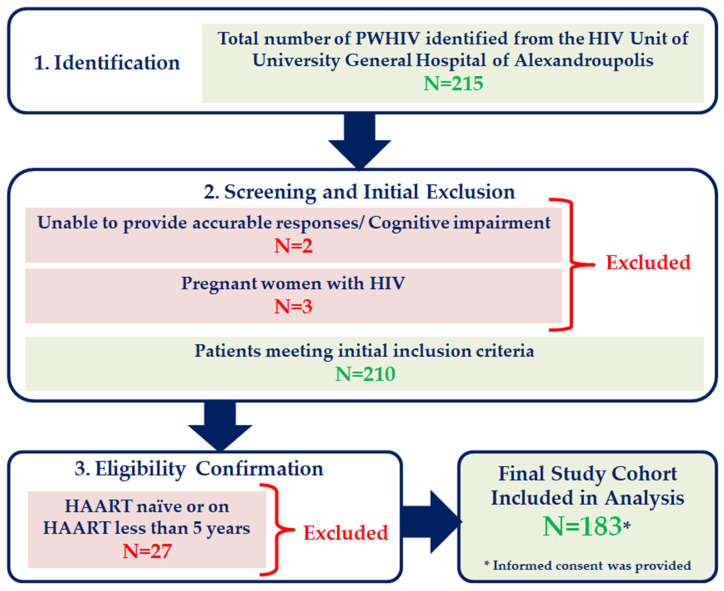
Flowchart of study participants’ selection.

This is an observational study with a cross-sectional design, conducted in the HIV Unit of University General Hospital of Alexandroupolis during the period from January 2024 to January 2025. We included patients diagnosed with HIV infection, whose age was ≥18 years old and were on HAART at least 5 years. The ≥5 years inclusion criterion was implemented to generate data relevant to the long-term clinical management of PWHIV by studying a cohort that has achieved viral suppression and faces the chronic burdens of cumulative disease and treatment exposure. Pregnant women with HIV, individuals unable to provide accurate responses due to cognitive impairment, and naïve patients or patients receiving HAART less than 5 years were excluded. The study was conducted following the Helsinki Declaration of research involving human subjects and written informed consent was obtained from all subjects. Flowchart of study participants’ selection is shown in Figure 1.

Sociodemographic characteristics (age, sex, education level, marital status) and lifestyle related factors (alcohol consumption, smoking habits, physical activity) were reported based on patient declarations via a structured interview conducted during the enrollment visit. Smoking status was categorized as current smoker, ex-smoker, or never smoker. Alcohol consumption was defined as self-reported regular consumption. Physical activity was classified as regular (defined as ≥150 min of moderate activity per week) or irregular/sedentary. All lifestyle data reflect the patient’s status at the time of the study visit. Body measurements were conducted including height and body mass index (BMI). Based on BMI values individuals were classified as underweight for BMI ≤ 18.5 kg/m^2^, normal for BMI between 18.5 and 24.9 kg/m^2^, overweight for BMI between 25.0 and 29.9 kg/m^2^, and obese for BMI > 30 kg/m^2^ [14]. HIV-specific data such as time of HIV diagnosis, CDC stage of HIV infection, HAART (agents, treatment compliance), CD4 cell count (at diagnosis, current value), HIV-RNA viral load and transmission mode, and coexisting comorbidities such as diabetes mellitus, malignancies, coronary disease, and respiratory problems were documented analyzing patient medical reports. The specific ART agents reported and used in the regression reflect the drug class present in the patient’s current, active HAART regimen at the time of study enrollment, given the cross-sectional design. HAART adherence was assessed using patient self-report during the structured interview. PWHIV were categorized into two groups based on adherence in the prior 12 months. Total compliance was defined as reporting 100% adherence (zero missed doses) and impartial adherence as reporting <100% adherence (one or more missed doses per month). In Greece there is no available laboratory for the measurement of serum levels of antiretroviral agents. HIV-RNA viral load was defined as detectable (≥50 copies/mL) based on laboratory records and represents the objective virologic outcome. This objective measure was analyzed independently of HAART adherence, which was assessed via patient self-report.

PWHIV with a prior diagnosis of hypertension, defined as any documented clinical diagnosis made and recorded before the initiation of the study period (January 2024), were classified as known cases. Their blood pressure was measured, and current antihypertensive treatment was recorded. For those without a known history of hypertension, HTN was defined based on two elevated blood pressure measurements taken across two separate visits. Blood pressure measurements were conducted using electronic brood pressure monitors. The participants were in a rested, seated position for a minimum of fifteen minutes without smoking or coffee drinking for at least one hour. Three measurements were taken at three-minute intervals and the average of the last two readings was calculated in order to determine the participant’s blood pressure. The hypertension was defined as office systolic blood pressure of ≥140 mmHg or diastolic blood pressure of ≥90 mmHg based on the 2024 Guidelines of the European Society of Cardiology (ESC) [15]. Confirmation is recommended with out-of-office measurements or at least one repeated office measurement at a subsequent visit [15]. All patients who met the criteria for hypertension (≥140/≥90 mmHg) on the initial visit were asked to return for a repeat office blood pressure measurement on a subsequent day to confirm the diagnosis, as recommended by the guidelines [15]. Only those patients whose elevated blood pressure persisted upon this second, subsequent measurement were classified as having a final diagnosis of hypertension in the cohort and included in the HTN group. All participants provided a one-time, random urine sample. Urinary creatinine and albumin were measured using an immunoturbidimetric assay. Albuminuria was defined using the sex-based albumin–creatinine ratio (ACR). Albuminuria was defined as a value greater than 355 mg/g for females versus a value greater than 250 mg/g for males [16]. While these cutoffs differ from the non-sex-adjusted 300 mg/g threshold used in certain KDIGO (Kidney Disease, Improving Global Outcomes) guidelines, they are employed here to account for physiological differences in urinary creatinine excretion [16]. Measurements for urinary creatinine and albumin were conducted in the University Laboratory Department of the University General Hospital of Alexandroupolis, where the patients were monitored. Urinary albumin was quantified using the automated immunoturbidimetric assay, Tina-quant Albumin Gen. 2 (Roche Diagnostics, Basel, Switzerland), performed on a Cobas Integra clinical chemistry analyzer (Roche Diagnostics, Basel, Switzerland). The instrument utilized software version v4.1 for data analysis.

Statistical analysis of the data was performed using IBM Statistical Package for the Social Sciences (SPSS), version 19.0 (IBM Corp., Armonk, NY, USA). The normality of quantitative variables was tested with Kolmogorov–Smirnov test. Normally distributed quantitative variables are expressed as the mean ± standard deviation (SD), while non-normally distributed quantitative variables are expressed as the median value and range. Qualitative variables were expressed as absolute and relative (%) frequencies. For the analysis of factors associated with the prevalence of hypertension and albuminuria, a comprehensive set of candidate variables was initially considered. This pool included demographic factors (age, sex, education level, marital status, origin), lifestyle factors (smoking status, alcohol consumption, physical activity, BMI category), HIV-specific factors (years since HIV diagnosis, years on HAART, CDC stage, CD4 cell count at diagnosis and current, detectable viral load, HAART adherence, transmission mode, and current ART regimen classes), and comorbidities. Each of these variables was first examined in univariate analysis for its association with the respective outcomes. Univariable analysis was performed using Student’s *t*-test, Mann–Whitney U-test, and chi-square test. Multivariable analysis was performed using a Modified Poisson Regression Model with a robust error variance to obtain adjusted Prevalence Ratios (aPR). Variables significant in the univariate analysis were entered into the final multivariable model, using a backward stepwise approach to identify factors independently associated with the prevalence of the outcomes. All tests were two tailed and statistical significance was considered for *p* values < 0.05.

## 3. Results

The study included 183 PWHIV with a mean age of 46.71 ± 11.73 years (Table 1). Consistent with HIV epidemiology in Greece, the cohort was predominantly male (72.7%) and MSM (59.7%). The majority were highly educated, with 85.3% having completed high school or university. Regarding HIV-specific characteristics, all patients had been on HAART for a mean of 5.8 ± 2.6 years, with 86.3% reporting total adherence. Current immunological control was robust, reflected by a median CD4 cell count of 585 cells/mm^3^ and only 9.8% having a detectable viral load. TAF-based regimens and integrase inhibitors were the most commonly used ART classes, reflecting current treatment guidelines. From a clinical and lifestyle perspective, notable 65.0% were current smokers, indicating a significant modifiable risk factor burden. BMI distribution showed a high prevalence of being overweight (43.2%) and obese (4.9%). Comorbidity burden was substantial, with 55.2% having at least one comorbidity and 40.4% having more than one. Dyslipidemia was highly prevalent (51.4%), with hypertension affecting 28.96% of known cases and diabetes mellitus present in 11.5% (Table 1). This profile underscores the growing challenge of managing non-AIDS-related conditions in this population.

**Figure 2:**
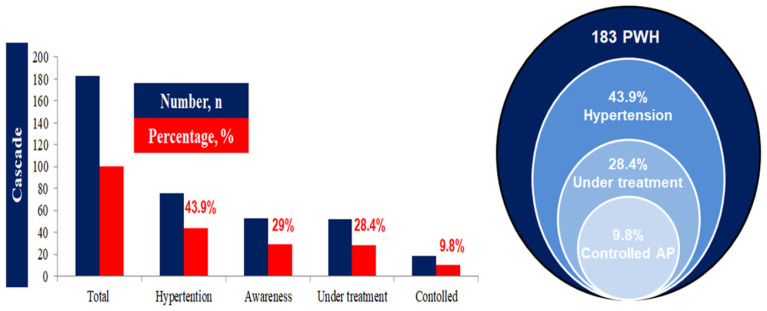
Cascade of hypertension in the study population with HIV.

Hypertension (HTN) was highly prevalent, diagnosed in 43.9% (*n* = 76) of the total study cohort (Figure 2). Of the 183 participants, 76 individuals were classified as having HIV and HTN, while 107 had HIV with normal blood pressure. Analysis of HTN management showed low rates of awareness and control. While 45 males and 8 females with HTN were aware of their diagnosis, and 44 males and 8 females were under treatment, only 15 males and 3 females achieved controlled blood pressure with treatment (Figure 2). Factors associated with the presence of HTN were assessed by comparing the 76 participants with HTN against the 107 normotensive participants. Univariate analysis identified several factors significantly associated with hypertension. Both being overweight (PR 2.45, *p* = 0.042) and obese (PR 4.50, *p* < 0.001) were strongly associated with HTN. The presence of at least one comorbidity (PR 1.80, *p* = 0.050), more than one comorbidity (PR 2.30, *p* < 0.001), and diabetes mellitus (PR 3.20, *p* < 0.001) were all statistically significant predictors (Table 2). In the final multivariable Modified Poisson Regression Model (Table 2), controlled for potential confounders, age (41–60 years, aPR 1.40; >60 years, aPR 2.00), being overweight (aPR2.30), and obesity (aPR4.20) remained powerful independent predictors. The analysis of potential associations between HIV data and the presence of HTN demonstrated that while HIV infection duration, CDC stage, and viral load were not significant independent predictors in the multivariable model, TDF-based regimens were statistically significant predictors (aPR 1.85, *p* = 0.065) (Table 3). The collinear variable, HIV infection duration, was excluded from the final multivariable model.

Table 4 presents the univariable comparison of demographic, clinical, and HIV-specific characteristics between patients with and without prevalent albuminuria. These comparisons highlight the crude differences in characteristics for the outcome of albuminuria, laying the groundwork for the subsequent multivariable analysis. Individuals with albuminuria were significantly older (mean age 54 years) and had a dramatically higher frequency of hypertension (total number of PWHIV with known HTN or diagnosed during the study), affecting 82.9% of these individuals compared to only 29.6% of PWHIV without albuminuria. Unadjusted prevalence ratios (PR) analysis (Table 5) identified multiple significant predictors of albuminuria. Diabetes mellitus had the highest prevalence ratio (PR = 8.65, *p* < 0.001), followed by hypertension (PR = 6.80, *p* < 0.001) and more than two comorbidities (PR = 2.36, *p* < 0.001). Each one-year increase in age (PR = 1.04) and duration on HAART (PR = 1.05) were also statistically significant predictors (*p* < 0.001). Conversely, male gender showed a statistically significant lower prevalence of albuminuria (PR = 0.65, *p* = 0.525) compared to females, but this association did not reach statistical significance. In the final adjusted model (aPR), duration on HAART (aPR 1.03 per year) remained a highly significant predictor alongside age and diabetes mellitus.

## 4. Discussion

This study, conducted in a cohort of PLWH characterized by high rates of complete HAART adherence and viral suppression, provides critical insights into the evolving landscape of non-AIDS related comorbidities, specifically focusing on the pathogenesis of HTN and albuminuria. Our findings confirm the shift in disease burden, demonstrating that in the era of effective HAART, traditional cardiovascular and metabolic risk factors are the dominant predictors of HTN, while HTN and other systemic comorbidities are strong risk factors associated with renal damage, as measured by albuminuria. The high prevalence of HTN in our cohort (43.9%) is consistent with published meta-analyses, particularly those focusing on ART-experienced populations, where prevalence rates approach 35% [17,18,19,20,21,22,23,24]. Notably, the multivariable analysis of HTN confirmed the strong independent predictive capacity of increased age, obesity, and dyslipidemia, while none of the studied HIV-specific parameters including CD4 cell count, viral load, or duration of the infection showed a significant association with HTN. However, the results regarding the albuminuria reveal a more complex interaction. While traditional factors like diabetes mellitus and age remain powerful predictors, the significant association between albuminuria and the duration of HAART exposure (aRR1.03 per year) suggests that either persistent, HIV-related inflammation or the cumulative effects of long-term ART may contribute to microvascular damage in the kidney. The collinear nature of HIV infection duration and HAART duration led us to exclude HIV infection duration from the final multivariable model (Table 5), leaving HAART duration as the most precise measure of long-term therapeutic exposure. This finding is consistent with studies that suggest long-term ART exposure, especially to nephrotoxic agents like TDF (a class used by 18% of our cohort), can impair kidney tubular function, leading to albuminuria [25,26,27,28,29,30,31,32].

Early epidemiological studies in previous decades suggested that the prevalence of hypertension in PWHIV is lower compared to general population without HIV infection due to high incidence of wasting syndrome, opportunistic infections, malignancies, and cachexia associated with advanced stages of HIV infection [26]. However, after widespread administration of HAART studies support that the hypothesis of ‘return to health’ and weight managed to reverse this trend [27]. Numerous studies presenting real-world data have consistently demonstrated a higher prevalence of hypertension in PWHIV, which varies across studies ranging from 20% to over 50% depending on cohort characteristics such as age, race, ethnicity, and the definition of hypertension [28,29,30,31]. This prevalence remains increased even after controlling traditional risk factors in PWHIV including body mass index, smoking, and diabetes mellitus [30]. The systematic review and meta-analysis by Xu et al. highlighted the varying and substantial prevalence by HTN across global studies involving PWHIV [32]. The overall pooled prevalence of HTN in the analysis which aggregated data from 49 studies and over 63.000 participants was estimated at 25.2% [32]. Crucially, the prevalence differed significantly based on the treatment status of the cohort and was markedly higher among participants who were ART-experienced, reaching 34.7% compared to a much lower prevalence of 12.7% in ART-naïve individuals. Furthermore, the study noted that HTN prevalence increased with the age of the cohort and was higher in studies conducted more recently, underscoring HTN as a growing and serious non-AIDS complication in the aging population of PWHIV [32].

Albuminuria is also a common finding affecting 10–30% of PWHIV [33,34,35,36]. The presence of microalbuminuria is a particularly strong predictor of the progression of kidney disease and cardiovascular events [37]. Studies have shown that PWHIV with albuminuria have a significantly higher risk of all-cause mortality and end-stage renal disease [38,39,40,41]. The combination of hypertension and albuminuria is particularly potent [42]. A meta-analysis of multiple cohorts found that PWHIV with both conditions had a dramatically higher risk for major adverse cardiovascular events compared to those with neither condition underlying the synergistic risk posed by these comorbidities [43].

The elevated rates of hypertension and albuminuria in PWHIV are not merely a consequence of aging, but they are driven by a unique set of mechanisms related to the chronic effects of HIV and HAART [44]. Even after effective viral suppression, a state of chronic low-grade inflammation and immune activation persists, driven by ongoing microbial translocation from the gut, residual viral replication in tissue reservoirs, and coinfections [45]. Chronic inflammation leads to endothelial dysfunction, a key upstream event in the development in both hypertension and kidney damage [45]. Inflammatory cytokines disrupt the normal function of endothelium impairing nitic oxide production and promoting vasoconstriction and elevation of blood pressure [46]. In the kidneys, chronic inflammation leads to glomerulosclerosis and interstitial fibrosis inducing a loss of glomerular filtration surface area and impaired tubular function, decreased filtration capacity, and increased leakage of albumin into the urine [4].

HIV can directly infect and replicate in carious cells, including the kidney podocytes and tubular epithelial cells which are crucial for maintaining the glomerular filtration barrier [47]. HIV-associated nephropathy (HIVAN), a district form of a collapsing focal segmental glomerulosclerosis, is less common in HAART era, but remains a significant cause of albuminuria and rapid progression to end-stage renal disease, particularly among individuals of African descent [48]. Certain classes of antiretrovirals have been implicated in the development or exacerbation of hypertension and albuminuria. Early protease inhibitors (PIs) were associated with metabolic complications including insulin resistance and dyslipidemia, which are known risk factors for hypertension and chronic cardiovascular disease [49]. Some PIs such as indinavir were also linked to crystalluria and nephrotoxicity [50]. Tenofovir Disoproxil Fumarate (TDF) is a cornerstone of HAART, but it is known that it could cause renal tubular dysfunction, leading to Fanconi syndrome, phosphaturia, and, in some cases, acute kidney injury [51]. This tubular damage can result in albuminuria even in the absence of a significant decline in glomerular filtration rate (GFR) [51]. While generally considered metabolically neutral, there is a number of studies which support that integrase strand transfer inhibitors (INSTIs) based regimens, particularly those containing dolutegravir, may be associated with weight gain and an increased risk of hypertension [52]. The exact mechanism remains unclear, but it is probably linked to changes in lipid metabolism and other systemic effects [52]. Studies have shown that PWHIV switching from efavirenz to dolutegravir (DTG) experienced greater weight gain and increased hypertension risk compared to those remaining on efavirenz due to DTG-associated weight gain [53]. INSTI use was associated with greater weight gain compared to PI or NNRTIs and this association was greater with the newer INSTIs (bictegravir and dolutegravir) compared to elvitegravir [54,55]. Findings from the RESPOND study indicated a higher association of hypertension with INSTI use compared with NNRTIs in both ART-naïve and ART-experienced participants [56].

Several studies have been conducted measuring the levels of inflammatory markers in plasma samples and showed that HIV-related inflammation is associated with a high prevalence of HTN, although another study in PWHIV did not find a link between the levels of inflammatory markers, such as IL-6 and hsCRP and hypertension [55,56,57,58]. PWHIV with severely exhausted (PD1+) CD4+ and CD8+ T cells seem to have stiffer arteries during the first 3 months of ART [59]. Certain antiretroviral classes, such as NNRTIs, traditional factors (age, higher body mass index), and the advanced stage of HIV infection, may also contribute to the development of hypertension [60,61]. A longitudinal study examining blood pressure changes in PWHIV on ART compared with people without HIV in rural Uganda showed lower blood pressure over 4 years, with a rise over time mostly mediated by increased body mass index, highlighting the effect of healthcare access and lifestyle differences [62]. Another study among virally suppressed PWHIV compared with people without HIV in rural South Africa indicated that engagement in HIV care was associated with improved hypertension care but worsened diabetes care [63]. Cross-sectional data from the WHO SAGE Well-Being of Older People Study found that more than 50% of older PWHIV in South Africa are hypertensive, but the mechanisms driving hypertension in PWHIV are not clear [64]. Microbial translocation, endothelial dysfunction, abnormal activation of the RAAS (renin–angiotensin–aldosterone system), renal disease, dyslipidemia, sympathetic activation, and immune reconstitution have been suggested as possible mechanisms [65,66]. There is evidence of RAAS dysregulation in PWHIV, and renin, angiotensin II, and aldosterone levels are elevated compared with people without HIV [61,62]. Chronic inflammatory condition directly impairs the renal microvasculature, resulting in a compromised glomerular filtration barrier and subsequent albuminuria [63,64]. Furthermore, the use of HAART is not without its metabolic and renal consequences while certain ART classes, such as NRTIs, have been associated with mitochondrial toxicity, which can impair kidney tubular function [65]. Protease inhibitors (PIs) are also linked to lipodystrophy, insulin resistance, and dyslipidemia, further exacerbating the risk of hypertension and renal injury [66]. Coinfections, such as hepatitis B and C viruses, frequently reported in PWH, can independently lead to chronic inflammation and kidney disease. Injecting drugs and lifestyle factors, such as high rates of smoking contribute to the elevated risk of hypertension and albuminuria [67]. Targeted prevention and management strategies are crucial to be developed in order to reduce the morbidity rates and improve the quality of life highlighting the need of a holistic approach of care beyond viral suppression [68]. Crucially, our data provide real-world support for the central premise of the REPRIEVE trial (phase 3 of Randomized Trial to Prevent Vascular Events in HIV), indicating that non-AIDS cardiovascular and renal risks remain high even in virally suppressed individuals, mandating comprehensive screening that addresses both traditional metabolic factors and cumulative ART exposure to effectively mitigate long-term morbidity [67].

The high prevalence and early onset of hypertension and albuminuria in PWHIV necessitate a proactive approach for screening and management [68]. A methodological strength in our study is that HTN diagnosis was confirmed on a second visit, aligning with the 2024 ESC Guidelines and strengthening the validity of our reported prevalence. Routine screening is vital for both conditions. Regular blood pressure measurements should be conducted at every clinical visit, and a spot urine albumin–creatinine ratio (UACR) should be measured at baseline and at least annually or more frequently in people with comorbidities such as diabetes mellitus or pre-existing renal dysfunction [68]. Initiating antihypertensive medications before experiencing a cardiovascular disease-related clinical event is associated with reduced risk of acute myocardial infraction, stroke, and death [67]. Microalbuminuria is a prevalent and clinically significant marker of cardiovascular risk in hypertensive patients and early detection is vital in order to identify high-risk individuals, manage risk factors, and prevent long-term complications [69]. The management of hypertension in PWHIV follows similar principles to the general population [69]. Regular physical activity, a low-sodium diet, moderation of alcohol intake, and smoking cessation are essential combined with pharmacotherapy [70]. Angiotensin-converting enzyme (ACE) inhibitors and angiotensin receptor blockers (ARBs) are first-line agents because they do not only lower blood pressure but also have potent renoprotective effects, particularly in the presence of albuminuria reducing intraglomerular pressure and decreasing albumin excretion [69].

The main limitation of this study is its cross-sectional design. While our findings demonstrate strong associations between traditional risk factors (age, BMI) and HAART duration with the prevalence of hypertension and albuminuria, this design limits the ability to establish causation or the precise temporal relationship between these factors and the onset or progression of cardiovascular and renal disease. The demographic composition of our study population represents a significant limitation on the generalizability of our findings. The cohort is predominantly male (72.7%), with the majority of transmissions occurring in MSM (59.7%). This weighing toward a specific demographic group means that our conclusions regarding the predictors of hypertension and albuminuria are primarily reflective of the clinical landscape within Greek MSM populations. Women with HIV often face different metabolic profiles, hormonal influences, and potentially different adverse event profiles related to ART (such as those concerning weight gain) compared to men [48,49,50,51,52,53,54]. Applying our PR estimates for conditions like dyslipidemia or obesity directly to women with HIV may therefore be inaccurate. While we used sex-based thresholds for defining albuminuria, the low representation of women (27.3%) limits the statistical power needed to identify unique, sex-specific risk factors. The low representation of heterosexual and PWID groups also limits the demonstration of associations to those populations.

## 5. Conclusions

The successful progression of HIV infection into a chronic, manageable condition has brought to the forefront the challenges of managing comorbidities. Hypertension and albuminuria are increasingly prevalent in the aging HIV population, acting as key drivers of cardiovascular and renal morbidity and mortality. Our data demonstrate that while traditional metabolic factors dominate HTN risk, factors such as age, diabetes mellitus, and the cumulative duration of HAART contribute to renal progression as measured by albuminuria. These findings underline the critical need for the screening and management of hypertension and other comorbidities to mitigate the risk of long-term cardiovascular and renal complications in this aging population of PWHIV.

## Figures and Tables

**Table 1:** Demographic, clinical, and HIV-specific characteristics of study population (*n* = 183).

Parameter		*n* = 183
Demographics		
Age, Mean Value ± SD (years)		46.71 ± 11.73
Sex, *n* (%)	Male	133 (72.67%)
	Female	50 (27.33%)
Origin, *n* (%)	Greek	165 (90.16%)
	Other	18 (9.84%)
Family status, *n* (%)	Unmarried	145 (79.23%)
	Married	38 (20.76%)
Education status, *n* (%)	Primary school graduate	27 (14.75%)
	High school graduate	86 (46.99%)
	University graduate	70 (38.25%)
Lifestyle Factors and Body Mass Index		
Smokers, *n* (%)		119 (65.02%)
Regular alcohol consumption	Yes	96 (52.45%)
	No	87 (47.54%)
Physical activity	Regular (≥150 min of moderate activity per week)	35 (19.13%)
	Irregular	148 (80.97%)
BMI category, *n* (%)	Underweight (<18.5 kg/m^2^)	9 (4.91%)
	Normal weight (18.5–24.9 kg/m^2^)	86 (46.99%)
	Overweight (24.9–29.9 kg/m^2^)	79 (43.16%)
	Obese (30–34.9 kg/m^2^)	9 (4.91%)
HIV-specific data		
Transmission mode, *n* (%)	Heterosexual	47 (25.68%)
	Men who have sex with men (MSM)	92 (50.27%)
	People who inject drugs (PWID)	15 (8.19%)
CDC stage of HIV infection, *n* (%)	A	108 (59.01%)
	Β	45 (24.59%)
	C	31 (16.93%)
Years since HIV diagnosis, mean value ± SD (years)		6.31 ± 1.73
Years on ART, mean value ± SD (years)		5.82 ± 2.64
CD4 cell count (CD4 cells/mm^3^, median)	At diagnosis	432.25
	Current	585.47
Detectable HIV-viral load, *n* (%)		18 (9.83%)
HAART agent, *n* (%)	TDF-based	33 (18.03%)
	TAF-based	141 (77.04%)
	Integrase inhibitor	139 (75.95%)
	Protease inhibitor	25 (13.66%)
	Non-nucleoside reverse transcriptase inhibitor	14 (7.65%)
HAART adherence	Total	158 (86.33%)
	Impartial	25 (13.66%)
Comorbidities, *n* (%)		
At least one comorbidity		101 (55.19%)
More than one comorbidity		74 (40.43%)
Hypertension		53 (28.96%)
Diabetes mellitus		21 (11.47%)
Dyslipidaemia		94 (51.36%)
Coronary disease		15 (8.19%)
Heart failure		9 (4.91%)
Lung disease		13 (7.10%)
Malignancies		11 (6.01%)
Others		18 (9.83%)

**Table 2:** Unadjusted and adjusted prevalence ratios (PR/aPR) for predictors of hypertension (HTN) in PWHIV (*N* = 183).

	People with HIV and Normal BP (*N* = 107)	People with HIV and HTN (*N* = 76)	Unadjusted Prevalence Ratio (PR)	Adjusted Prevalence Ratio (aPR)
Variable	*N*	*N*	PR (95% CI)/*p*-Value	aPR (95% CI)/*p*-Value
Gender				
Male (Ref: Female)	76	57	1.77 (1.50, 2.10)/<0.001	1.70 (1.45, 2.05)/<0.001
Age Group				
20–40 years (Ref)	22	8	1.0/−	1.0/−
41–60 years	37	28	1.45 (1.10, 1.80)/<0.001	1.40 (1.05, 1.75)/<0.001
>60 years	48	40	2.05 (1.55, 2.60)/<0.001	2.00 (1.45, 2.50)/<0.001
Smoking				
No (Ref)	29	21	1.0/−	1.0/−
Yes	78	55	1.55 (1.30, 1.85)/<0.001	1.50 (1.25, 1.80)/<0.001
Alcohol consumption				
No (Ref)	52	35	1.0/−	1.0/−
Yes	55	41	1.45 (1.15, 1.75)/<0.001	1.40 (1.10, 1.70)/<0.001
Regular physical activity				
No (Ref)	82	66	1.0/−	1.0/−
Yes	25	10	1.52 (1.23, 1.68)/<0.001	1.36 (1.15, 1.82)/<0.001
BMI Category				
Normal (≤24.9)	65	30	1.0/−	1.0/−
Overweight	42	37	2.45 (1.80, 3.20)/0.042	2.30 (1.65, 3.00)/0.039
Obese	0	9	4.50 (3.00, 6.50)/<0.001	4.20 (2.85, 6.00)/<0.001
Comorbidities				
None (Ref)	60	22	1.0/−	1.0/−
At least one	47	54	1.80 (1.35, 2.40)/0.050	1.75 (1.30, 2.35)/0.050
More than one	31	46	2.30 (1.70, 3.10)/<0.001	2.20 (1.65, 3.00)/<0.001
Diabetes mellitus	5	16	3.20 (2.00, 5.00)/<0.001	3.00 (1.90, 4.80)/<0.001
Dyslipidemia	67	37	1.20 (0.95, 1.50)/0.065	1.15 (0.90, 1.45)/0.057
Coronary disease	6	9	1.50 (0.80, 2.80)/0.375	1.40 (0.75, 2.70)/0.482

**Table 3:** Unadjusted and adjusted prevalence ratios (PR/aPR) for HIV-specific data as predictors of hypertension (HTN) in PWHIV. LLV: low-level viremia is defined as HIV-1 plasma viral load above the limit of quantification of clinical assays (20–50 copies/mL), usually between 20 and 500 copies/mL, TDF: Tenofovir Disoproxil Fumarate, TAF: Tenofovir Alafenamide Fumarate.

	People with HIV and Normal BP (*N* = 107)	People with HIV and HTN (*N* = 76)	Unadjusted Prevalence Ratio (PR)	Adjusted Prevalnce Ratio (aPR)
Variable	*N*	*N*	pR (95% CI)/*p*-Value	aPR (95% CI)/*p*-Value
Years diagnosed with HIV				
<5 years (Ref)	25	12	1.0/−	1.0/−
5–9 years	27	29	1.24 (0.90, 1.50)/0.547	1.27 (0.60, 1.57)/0.075
>9 years	55	35	1.10 (0.90, 1.30)/0.487	1.15 (0.80, 1.34)/0.088
Years on HAART				
<5 years (Ref)	23	12	1.0/−	1.0/−
5–9 years	51	42	1.66 (1.20, 2.45)/0.037	1.87 (1.10, 3.53)/0.048
>9 years	33	22	2.40 (1.20, 4.10)/<0.001	2.35 (1.80, 4.10)/<0.001
CDC stage of HIV infection				
A (Ref)	59	10	1.0/−	1.0/−
B	32	32	1.20 (0.80, 1.45)/0.458	1.10 (0.90, 1.20)/0.450
C	16	34	1.70 (1.20, 2.40)/0.652	1.40 (1.20, 1.78)/0.524
Viral Load				
Undetectable (Ref)	85	46	1.0/−	1.0/−
Detectable	7	11	3.50 (2.50, 5.00)/<0.001	3.30 (2.30, 4.70)/<0.001
Low level viremia (LLV)	15	9	2.00 (1.20, 3.30)/0.004	1.80 (1.09, 3.00)/0.035
HAART Class				
Non-nucleoside reverse transcriptase inhibitor (Ref)	10	4	1.0/−	1.0/−
TDF-based	13	10	1.90 (0.95, 3.80)/0.055	1.85 (0.90, 3.70)/0.065
TAF-based	105	36	0.90 (0.75, 1.10)/0.455	0.95 (0.80, 1.15)/0.078
Integrase inhibitor	78	51	1.30 (0.75, 2.25)/0.405	1.25 (0.70, 2.10)/0.500
Protease inhibitor	15	10	1.50 (0.80, 2.80)/0.190	1.45 (0.75, 2.70)/0.250
Current CD4 cell count (cells/mm^3^)				
>500 (Ref)	42	31	1.0/−	1.0/−
<500	65	45	1.50 (1.10, 2.05)/<0.001	1.45 (1.05, 1.95)/<0.001

**Table 4:** Baseline characteristics of study population stratified based on the presence of albuminuria.

Demographic and Clinical Characteristics	Non-Albuminuria *n* = 142 (77.6%)	Albuminuria *n* = 41 (22.4%)	*p* Value
Age, years (SD)	49.22 (12.14)	54.02 (12.34)	<0.001
Females, *n* (%)	36 (25.35)	14 (34.15)	<0.001
Males, *n* (%)	106 (74.65)	27 (65.85)	
Overweight—obesity, *n* (%)	65 (45.77)	23 (56.09)	<0.001
Diabetes mellitus, *n* (%)	6 (4.25)	15 (36.58)	<0.001
Hypertension, *n* (%)	42 (29.57)	34 (82.92)	<0.001
Chronic kidney disease, *n* (%)	4 (2.81)	6 (14.63)	0.578
At least one comorbidity, *n* (%)	63 (44.36)	38 (92.68)	<0.001
More than one comorbidity, *n* (%)	44 (30.98)	30 (73.17)	<0.001
Systolic BP, mmHg (SD)	126.01 (15.91)	136.02 (19.41)	<0.001
Diastolic BP, mmHg (SD)	81.08 (9.25)	87.24 (12.92)	<0.001
HIV duration, years (SD)	6.13 (4.52–8.73)	6.32 (4.92–8.54)	0.587
ART duration, years (SD)	5.41 (4.22–7.91)	5.72 (4.21–8.23)	0.328

**Table 5:** Unadjusted prevalence ratios (PR), 95% confidence intervals (CI), and *p* values for demographic and clinical characteristics as risk predictors of albuminuria.

Clinical Factors	All Participants		Males		Females	
	PR (95% CI)	*p* Value	PR (95% CI)	*p* Value	PR (95% CI)	*p* Value
Age (per 1-year increase)	1.04 (1.02, 1.06)	<0.001	1.05 (1.03, 1.07)	<0.001	1.04 (1.01, 1.07)	0.009
Males	0.65 (0.45, 1.33)	0.525	-	-	-	-
Diabetes mellitus	8.65 (4.80, 11.75)	<0.001	8.15 (6.85, 11.82)	<0.001	7.62 (5.50, 11.15)	<0.001
Hypertension	6.80 (2.05, 8.82)	<0.001	7.05 (4.10, 8.90)	<0.001	3.65 (2.70, 6.10)	<0.001
More than two comorbidities	2.36 (1.60, 3.30)	<0.001	2.50 (1.65, 3.80)	<0.001	2.00 (1.00, 4.00)	0.050
Systolic blood pressure (per 2 mmHg increment)	1.04 (1.02, 1.06)	<0.001	1.03 (1.01, 1.05)	0.003	1.05 (1.02, 1.08)	0.001
Diastolic blood pressure (per 2 mmHg increment)	1.03 (1.01, 1.05)	<0.001	1.02 (1.01, 1.03)	0.005	1.04 (1.02, 1.06)	0.002
Duration of HAART (per 1-year increase)	1.05 (1.03, 1.07)	<0.001	1.04 (1.02, 1.06)	<0.001	1.06 (1.03, 1.09)	<0.001

## Data Availability

Our research data are available after a request to the corresponding author.
